# Georgia Medical Association

**Published:** 1876-05

**Authors:** 


*Georgia Medical Association.*—Circumstances prevented our attendance at the last meeting of the Georgia Medical Association, which assembled at Augusta, on the 19th inst. We submit brief abstracts of the proceedings:

The Association was called to order by President Ford. An address of welcome was made by Dr. H. F. Campbell, and responded to by Dr. Johnson, of Atlanta.

The President elect, Dr. J. G. Thomas, addressed the Association on the objects and benefits conferred by the State Board of Health.

Several interesting papers were read on various subjects.

A committee consisting of Drs. E. J. Kirkscey, J. M. Johnson, T. J. Word, G. E. Sussdorf and G. J. Grimes were appointed, to report at next meeting "On the Rise and Progress of Medicine in Georgia for the past hundred years."

Delegates appointed to the American Medical Association, (to convene in Philadelphia, January 6th): Drs. J. P. Logan, T. S. Hopkins, G. M. McDowell, J. G. Westmoreland, L. A. Dugas, J. T. Johnson, H. F. Scott, K. P. Moore, J. C. LeHardy, DeSaussure Ford, Juriah Harris, R. J. Nunn, R. H. Lewis, G. F. Cooper, B. M. Cromwell, W. A. Green, G. E. Sussdorf, E. L. Crump, W. S. Kendrick, R. M. Smith, T. J. Word, R. C. Eve, W. F. Holt, J. S. Coleman, Eugene Foster, E. W. Alfriend, A. W. Calhoun, S. G. White, E. J. Kirkscey, R. F. Wright, Sterling Eve, L. D. Ford, J. T. Banks, V. H. Taliaferro, W. A. Love and R. D. Doster.

Delegates to the International Medical Congress, to meet in Philadelphia, September 4th: Drs. J. A. Eve, Robt. Battey, R. J. Nunn, W. H. Doughty, W. F. Westmoreland, D. W. Hammond, H. F. Campbell, F. A. Standford, W. O'Daniel and J. M. Johnson.

Amendments to the By-Laws were made fixing the initiation fee and the annual assessment at five dollars; failure to pay for two consecutive years forfeiting membership.

Officers elected for next term:

For President—Dr. Robert Battey.

For 1st Vice President—Dr. K. P. Moore.

For 2nd Vice President—Dr. A. W. Calhoun.

For Censor—Dr. R. M. Smith.

Dr. A. S. Campbell, orator, made the Annual Address on Thursday night.

Macon was chosen as the place for the next annual meeting.

The following committees were appointed:

Committee of Arrangements—Drs. Sussdorf, Hall, Burgess, Holt, and Wright.

Committee on Publication—Drs. J. T. Johnson, W. O'Daniel, J. B. Baird, W. S. Armstrong and W. S. Kendrick.

Committee on Necrology—Drs. J. M. Johnson, J. C. Blackshear, J. C. LeHardy, J. C. Bacon, R. C. Eve.

*Congressional Sections.*—First District—Practice of Medicine—Drs. J. G. Thomas, B. L. Crump, B. S. Purse. Surgery—T. J. Charlton, W. G. Bullock, R. P. Myers. Gynæcology—J. C. LeHardy, T. Smith, L. B. Bouchelle.

Second District—Practice of Medicine—P. L. Hillsman, R. B. Doster, W. M. Bruce. Surgery—R. T. Kendrick, W. J. Harrell, S. W. Burney. Gynæcology—J. A. Butts, D. S. Brandon, E. W. Alfriend.

Third District—Practice of Medicine—S. B. Hawkins, J. H. Stephens. J. W. Tucker. Surgery—H. V. Johnson, T. F. Walker, J. B. Hinkle. Gynæcology—W. J. Reese, Y. H. Morgan, G. F. Cooper.

Fourth District—Practice of Medicine—T. F. Brewster, E. L. Bardwell, C. B. Leitner. Surgery—A. B. Calhoun, Geo. Grimes, F. L. Wisdom. Gynæcology—F. A. Stanford, J. E. Bacon, T. J. Word.

Fifth District—Practice of Medicine—J. T. Banks, R. B. Murchison, John G. Westmoreland. Surgery—W. F. Westmoreland, C. S. Strother, A. W. Calhoun. Gynæcology—T. S. Powell, Paul Faver, R. C. Word.

Sixth District—Practice of Medicine—W. R. Burgess, C. H. Hall, W. A. Greene. Surgery—D. W. Hammond, W. O'Daniel, G. E. Sussdorf. Gynæcology—T. H. Kenan, A. Kingman, J. E. Blackshear.

Seventh District—Practice of Medicine—J. B. Underwood, W. D. Hoyt, William Farrell. Surgery—C. P. Gordon, J. B. S. Holmes, M. W. Gray. Gynæcology—Robert Battey, W. P. Harden, J. M. Gregory.

Eighth District—Practice of Medicine—E. H. Hunter, Eugene Foster, W. W. Battey. Surgery—L. A. Dugas, DeSaussure Ford, A. S. Campbell. Gynæcology—J. A. Eve, J. S. Coleman, H. F. Campbell.

Ninth District—Practice of Medicine—W. T. Hollingsworth, C. W. Long, J. E. Pope. Surgery—A. J. Shaffer, J. W. Bailey, R. M. Smith. Gynæcology—J. B. Carlton, J. S. Simmons, H. R. J. Long.

The Board of Censors made a report disclaiming any intention of reflecting on any member of the Middle Georgia Medical Society in their report of last year.

A banquet was given the members on Thursday, and a generous hospitality was extended to them by the citizens and physicians of Augusta.

For the Southern Medical Record.]

